# Picture Fuzzy Threshold Graphs with Application in Medicine Replenishment

**DOI:** 10.3390/e24050658

**Published:** 2022-05-07

**Authors:** Sankar Das, Ganesh Ghorai, Qin Xin

**Affiliations:** 1 Department of Applied Mathematics with Oceanology and Computer Programming, Vidyasagar University, Midnapore 721102, India; sankarkgp22@gmail.com; 2 Department of Mathematics, Kharagpur College, Kharagpur 721305, India; 3 Faculty of Science and Technology, University of the Faroe Islands, 100 Torshavn, Faroe Islands; qinx@setur.fo

**Keywords:** picture fuzzy graph, picture fuzzy threshold graph, picture fuzzy split graph, picture fuzzy threshold dimension, picture fuzzy partition number

## Abstract

In this study, a novel concept of picture fuzzy threshold graph (PFTG) is introduced. It has been shown that PFTGs are free from alternating 4-cycle and it can be constructed by repeatedly adding a dominating or an isolated node. Several properties about PFTGs are discussed and obtained the results that every picture fuzzy graph (PFG) is equivalent to a PFTG under certain conditions. Also, the underlying crisp graph (UCG) of PFTG is a split graph (SG), and conversely, a given SG can be applied to constitute a PFTG. A PFTG can be decomposed in a unique way and it generates three distinct fuzzy threshold graphs (FTGs). Furthermore, two important parameters i.e., picture fuzzy (PF) threshold dimension (TD) and PF partition number (PN) of PFGs are defined. Several properties on TD and PN have also been discussed. Lastly, an application of these developed results are presented in controlling medicine resources.

## 1. Introduction

Graphs can be considered as the bonding of objects. To emphasis on a real problem, those objects are being bonded by some relations such as, friendship is the bonding of pupil. If the vagueness in bonding arises, then the corresponding graph can be modelled as fuzzy graph (FG) model. In twenty first century, the graph theory has been fully exploited by fuzzy theory. In a crisp graphs two vertices are either related or not related to each other, mathematically, the degree of relationship is either 0 or 1. While in FGs, the degree of relationship takes values from [0,1]. The concept of FG, intuitionistic FGs and their extensions such interval valued FGs and interval valued intuitionistic FGs, and so on, have been studied deeply in over hundred papers. All these types of graphs have a common property that each edge must have a membership value less than or equal to the minimum membership of the nodes it connects. Famous Mathematician Euler described the solution of the seven bridge problem in 1736. It has become conventional to preserve the application of graph theory in different situations such as computer network, electric network, etc. In the literature, threshold graphs (TGs) as special graphs having beautiful structures and several important mathematical properties. It has a large impact in graph theory (GT) as well as in many applied areas, such as psychology, artificial intelligence, computer science, etc. These graphs can also be used to control flow of information between processors, similar to how traffic lights are used in controlling the flow of traffic.

In existing papers on TGs, all information were collected in fuzzy sense. The FTG or intuitionistic FTG model, usually demonstrate the information having fuzzy in nature. But, when description of the object or their relations or both is indeterminate and inconsistent, it cannot be handled by existing TGs models. In these scenario, the information should be taken in picture fuzzy sense. For this purpose, we proposed the concept of PFTGs based on literal indeterminacy to deal with such situations, which is more precise and more flexible than the other existing models, where vagueness and uncertainty will be characterized by picture fuzzy sets (PFSs) instead of fuzzy sets and intuitionistic fuzzy (IF) sets. However, there are are some unsolved issues in the literature:

(1) The TG is an advance studied topic and it was discussed in several fields except in PF field.
(2) The crisp TG, FTG and intuitionistic FTG models do not recognize all real life systems having an indeterminate information.
(3) The PFTG models are more helpful to control the flow of information than the other existing models. But, till now it was not formulate in PF environment.

The use of PFTG model in controlling several resources, which have not been addressed in the literature. In this paper, it has been shown that PFTG model can be used to control medicine resource. This study answers the following research questions:

(1) Is it possible to formulate a new TG model can help to solve resources allocation problems in PF surroundings?
(2) Is it possible to handle the systems having an indeterminate information by using PFTG models?

The FTG [1] or intuitionistic FTG [2] model, generally exhibit the system having fuzzy in nature. But, when its objects have more uncertainties, then these models are unable to handle the system properly. In such circumstances, PFTG models are becoming useful because of their aim of reducing the differences between the traditional numerical models used to control resource allocation problems. Considering PFSs are more suitable and powerful to deal with uncertainty and vagueness compared to fuzzy sets and IF sets, PF models can give more precision and compatibility than fuzzy models in many applications. Motivated by the above analysis, it is really significant for us to study PFTGs compared to FTGs or intuitionistic FTGs, where vagueness and uncertainty will be characterized by PFSs [3] instead of fuzzy sets [4] and IF sets [5]. This study develops many novel observations in PF environment. The main Objectives of this paper are stated as follows:

(1) To extend the concept of FTG and intuitionistic FTG to PFTG to model systems having an indeterminate information.
(2) To fill the research gap, we propose TG models under PF environment.
(3) To compare the obtained results derived from the proposed model with the existing models.
(4) To control medicine resources using the proposed models.

The proposed model can be effective to control resources under PF environment. The theoretical contribution of this paper can be summarized as follows:

(1) We formulate PFTG along with its many interesting properties and then initiate the notions of picture fuzzy alternating 4-cycle (PFA4-C), TD and PN of PFGs.
(2) We introduce the relation of threshold values (TV) and vertex cardinality of a PFTG. We can decompose a PFTG in a unique way and it generates 3 distinct FTGs.
(3) We establish a relation between picture fuzzy TD and PN, and illustrates many important properties on decomposed FTG.
(4) We present the comparison of proposed TGs with the existing TGs.
(5) Finally, an application of PFTG is present in medicine replenishment problem. In this way, the research gap mentioned above can be filled.

The current work is novel in the sense that:

(1) It is capable to recognize all real life systems having an indeterminate information.
(2) It is more efficient and effective than in other field.
(3) It is based to control resources rather than in other existing TG models.

The rest of the paper is organized as follows: Section 2, makes a literature review on TGs. In Section 3, we describe some basic observations connected to PFTGs. Section 4, presents the conception of PFTG along with some important properties. Also, picture fuzzy TD and PN are introduced and a relation between them is establish. Some properties of TD and PN on decomposed FTG are also illustrates. Section 5, an application of PFTG in medicine replenishment problem is given. Before the concluding section, comparison of proposed TGs with the existing TGs are given in Section 6. Lastly, in Section 7, we present conclusion and future scope along with some limitations of the proposed method.

## 2. Related Works

The authors’ contribution to develop threshold graphs are discussed as follows- In 1973, the notion of TG was initially posed by Chvatal and Hammer [6] on set-packing problems. Ordman studied on threshold covering and resource allocation problems in [7]. Bipartite TGs were discussed by Hammer et al. in [8]. Next, Pedled and Mahadev [9] described some related topics on TGs. Tao et al. [10] introduced image thresholding by using graph cuts. Some notes on TGs had been studied by Andelic and Simic [11]. The concept of FTGs was first initiated by Samanta and Pal [1]. Next, Makwana et al. [12] exhibited the extraction of illumination invariant features appling fuzzy thresholding based approach. Later on, Pramanik et al. [13] presented an interval-valued FTG. Calamoneri et al. studied on dynamic TGs and their related classes in [14]. Mixed range-valued FTG colouring were studied by Jaculin and Srividhya [15] in 2018 and then, Yang and Mao [2] presented intuitionistic FTGs with their applications in controlling power resource and water resource. Recently, Mahapatra and Pal [16] discussed about *m*-polar FTG and implemented it in power resource controlling system. Next, Hameed et al. introduced extension of TGs in complex fuzzy field [17] and also in complex IF field [18]. Moreover, Akram et al. [19] exhibited TGs under picture Dombi fuzzy information and applied it to distribution of coal resources. Later on, they presented complex pythagorean FTGs with application in petroleum replenishment in [20].

In 2014, Cuong [3] was first grounded the conception of PFS theory, as a generalization of fuzzy set and IF set from philosophical point of view by incorporating the degree of indeterminacy or neutrality as independent component to deal with problems involving imprecise, indeterminate and inconsistent information. Then, Son [21] exhibited generalized picture distance measure with infliction in PF clustering and also narrated few measuring analogousness on PFSs [22]. Thong and Son discussed PF clustering: a new computational intelligence method [23] and PF clustering for complex data [24].

The concept of PFG was initiated by Al-Hawary et al. [25] with few operations. Next, Zuo et al. described new conceptions of PFGs in [26]. Mohamedlsmayil and AshaBosely [27] presented domination in PFGs. Later on, Xiao et al. [28] worked over regular PFG with infliction in communication systems. Next, Das and Ghorai [29] exhibited PF planar graphs and implemented it to design road network. They utilized the idea of PFSs to competition graphs, introduced the notion of PF competition graphs and implemented it in medical science in [30]. Also, the concept of *m*-step PF competition graphs was proposed by Das et al. [31] with their applications in education system, ecosystem and job competition. After that, they worked on the embedding of PFGs in various surfaces based on genus values and applying it to design electronic circuits in [32]. They also studied on PF tolerance graphs with application in sports competition in [33]. Next, Amanathulla et al. [34] presented balanced PFG and applied it to business alliance problem.

In this study, we initiated the notion of PFTG along with its several important properties and then presented the concepts of PFA4-C, TD and PN of PFGs. We introduced the relation of TV and vertex cardinality of a PFTG. We can decompose a PFTG in a unique way and it generates 3 distinct FTGs. Then we established a relation between picture fuzzy TD and picture fuzzy PN, and studied many important properties on decomposed FTG. We present the comparison of proposed TGs with the existing TGs. Finally, an application of PFTG is presented in medicine replenishment problem.

## 3. Preliminaries

Here, We reminisce few definitions those are connected to PFTG. TGs have a beautiful shapes and possess several significant mathematical properties. It plays a vital role in GT and also in other areas, such as artificial intelligence, psychology, computer science etc.

**Definition** **1.** *[6] A graph G=(V,E) is a TG if* ∃ *non-negative reals μ(p),(p∈V) and t such that $\sum_{p\in Ⓢ}\mu \left(p\right)\leq t$ iff Ⓢ(⊆V) is a stable set (SS) in G.*

Applying fuzzyness in GT, Rosenfeld [35] initiated concept of fuzzy graphs (FGs).

**Definition** **2.** *[35] A FG G=(V,σ,μ) is a triplet with V as node set, σ:V→[0,1] and μ:V×V→[0,1] are the degree of membership of b∈V and edge (b,c)∈G, respectively with μ(b,c)≤min(σ(b),σ(c))* ∀ *b,c∈V.*

**Definition** **3.** *[1] A FG G=(V,σ,μ) is FTG if* ∃ *non-negative reals σ(p),(p∈V) and t such that $\sum_{p\in Ⓢ}\sigma \left(p\right)\leq t$ iff Ⓢ(⊆V) is SS in G.*

PFS enhance the field of uncertain information.

**Definition** **4.** *[3] A PFS A is defined on an universe X as $A=\{d,\left(\mu_{A}\left(d\right),\eta_{A}\left(d\right),\nu_{A}\left(d\right)\right):d\in X\}$, where $\mu_{A}\left(d\right),\eta_{A}\left(d\right),\nu_{A}\left(d\right)\in \left[0,1\right]$ represent the degree of truth membership (TMS), abstinence membership (AMS), false membership (FMS) of d∈A, respectively with $0\leq \mu_{A}\left(d\right)+\eta_{A}\left(d\right)+\nu_{A}\left(d\right)\leq 1$ ∀d∈X. Also ∀d∈X, $D_{A}\left(d\right)=1-\left(\mu_{A}\left(d\right)+\eta_{A}\left(d\right)+\nu_{A}\left(d\right)\right)$ denote the denial degree of d∈A.*

PFG models gives more suitability and flexibility than the models in other fields.

**Definition** **5.** *[25] A PFG is G=(V,A,B) where $A=(\mu_{A},\eta_{A},\nu_{A})$, $B=(\mu_{B},\eta_{B},\nu_{B})$ and*

*(i) $V=\{p_{1},p_{2},\ldots ,p_{n}\}$ such that $\mu_{A},\eta_{A},\nu_{A}:V\to \left[0,1\right]$ are the degree of TMS, AMS and FMS of $p_{i}\in V$, respectively with $0\leq \mu_{A}\left(p_{i}\right)+\eta_{A}\left(p_{i}\right)+\nu_{A}\left(p_{i}\right)\leq 1$ $\forall p_{i}\in V$, (i=1,2,…,n).*

*(ii) $\mu_{B},\eta_{B},\nu_{B}:V\times V\to \left[0,1\right]$ are the degree of TMS, AMS and FMS of arc $\left(p_{i},p_{j}\right)$, respectively with $\mu_{B}\left(p_{i},p_{j}\right)\leq min\left\{\mu_{A}\left(p_{i}\right),\mu_{A}\left(p_{j}\right)\right\},$ $\eta_{B}\left(p_{i},p_{j}\right)\leq min\left\{\eta_{A}\left(p_{i}\right),\eta_{A}\left(p_{j}\right)\right\}$ and $\nu_{B}\left(p_{i},p_{j}\right)\leq max\left\{\nu_{A}\left(p_{i}\right),\nu_{A}\left(p_{j}\right)\right\}$ with $0\leq \mu_{B}\left(p_{i},p_{j}\right)+\eta_{B}\left(p_{i},p_{j}\right)+\nu_{B}\left(p_{i},p_{j}\right)\leq 1$∀$\left(p_{i},p_{j}\right)$.*

**Figure 1:**
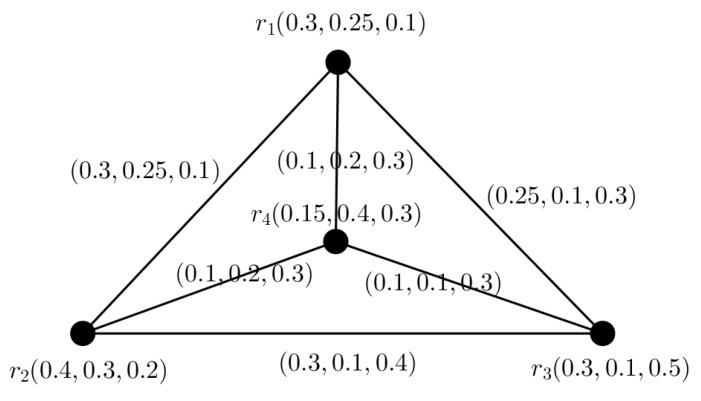
Example of a PFG.

**Example** **1.** *Consider the PFG G=(V,A,B) as showing in Figure 1, with $V=\{r_{1},r_{2},r_{3},r_{4}\}$, $A=\{\left(r_{1},\left(0.3,0.25,0.1\right)\right),\left(r_{2},\left(0.4,0.3,0.2\right)\right),\left(r_{3},\left(0.3,0.1,0.5\right)\right),\left(r_{4},\left(0.15,0.4,0.3\right)\right)\}$ is the PFS on V and $B=\{\left(r_{1}r_{2},\left(0.3,0.25,0.1\right)\right),\left(r_{1}r_{4},\left(0.1,0.2,0.3\right)\right),\left(r_{2}r_{3},\left(0.3,0.1,0.4\right)\right),\left(r_{2}r_{4},\left(0.1,0.2,0.3\right)\right),\left(r_{3}r_{4},\left(0.1,0.1,0.3\right)\right)\}$ is a PF relation on the PF subset of V×V. The TMS, AMS and FMS of the node $r_{1}$ are respectively 0.3, 0.25 and 0.1. Similarly for other nodes and arcs have.*

**Definition** **6.** *[27] A PFG G is complete if $\mu_{B}\left(a,c\right)=\mu_{A}\left(a\right)\wedge \mu_{A}\left(c\right),\eta_{B}\left(a,c\right)=\eta_{A}\left(a\right)\wedge \eta_{A}\left(c\right)$ and $\nu_{B}\left(a,c\right)=\nu_{A}\left(a\right)\vee \nu_{A}\left(c\right)$* ∀*a,c∈V.*

## 4. Picture Fuzzy Threshold Graph

Here, a new concept on PFG called PFTG is defined and exhibited few interesting properties of it.

**Definition** **7.** *A PFG G=(V,A,B) is called a PFTG with threshold $t=(t_{1},t_{2},t_{3})$, where $t_{1},t_{2},t_{3}\geq 0$ such that $\sum_{p\in Ⓢ}\mu_{A}\left(p\right)\leq t_{1}$, $\sum_{p\in Ⓢ}\eta_{A}\left(p\right)\leq t_{2}$ and $\sum_{p\in Ⓢ}\nu_{A}\left(p\right)\leq t_{3}$ iff Ⓢ(⊆V) is a SS in G. For simplicity, this PFTG is denote as $G=(A,B;t_{1},t_{2},t_{3})$.*

**Figure 2:**
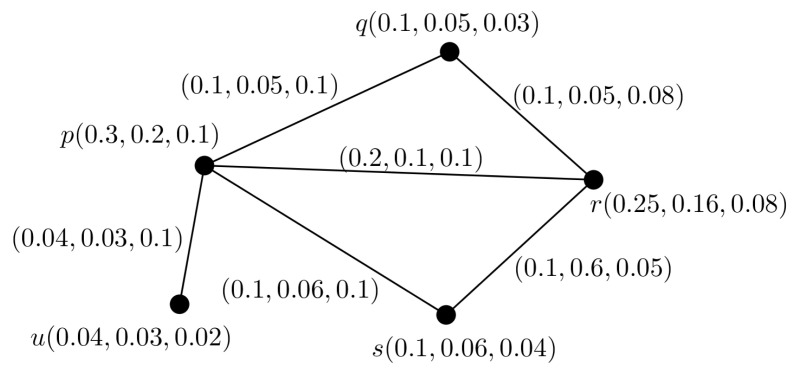
Example of a PFTG.

**Example** **2.** *We consider a PFG shown in Figure 2. Here one of the SS is Ⓢ={u,q,s} and for the SS Ⓢ, we have*

$\sum_{p\in Ⓢ}\mu_{A}\left(p\right)=0.04+0.1+0.1=0.24\leq 0.3$

$\sum_{p\in Ⓢ}\eta_{A}\left(p\right)=0.03+0.05+0.06=0.14\leq 0.2$

*$\sum_{p\in Ⓢ}\nu_{A}\left(p\right)=0.02+0.03+0.04=0.09\leq 0.1$.*

*Again, for any two non-SSs ${Ⓢ}_{1}=\left\{p,q,r\right\}$ and ${Ⓢ}_{2}=\left\{p,r,s\right\}$, we have*

$\sum_{p\in {Ⓢ}_{1}}\mu_{A}\left(p\right)=0.3+0.1+0.25=0.65>0.3$

$\sum_{p\in {Ⓢ}_{1}}\eta_{A}\left(p\right)=0.2+0.05+0.16=0.41>0.2$

*$\sum_{p\in {Ⓢ}_{1}}\nu_{A}\left(p\right)=0.1+0.03+0.08=0.21>0.1$ and*

$\sum_{p\in {Ⓢ}_{2}}\mu_{A}\left(p\right)=0.3+0.25+0.1=0.65>0.3$

$\sum_{p\in {Ⓢ}_{2}}\eta_{A}\left(p\right)=0.2+0.16+0.06=0.42>0.2$

*$\sum_{p\in {Ⓢ}_{2}}\nu_{A}\left(p\right)=0.1+0.08+0.04=0.22>0.1$.*

*So, the PFG shown in Figure 2 is a PFTG G=(A,B;0.3,0.2,0.1).*

**Definition** **8.** *A PFG G=(V,A,B) with 4 vertices is a PF square $C_{4}$ graph if its vertices can be label $v_{1},v_{2},v_{3},v_{4}$ such that $\left(\left(v_{i},v_{i}+1\right),\mu_{B},\eta_{B},\nu_{B}\right)\neq \left(0,0,0\right)$, 1≤i≤3 with $v_{1}=v_{4}$.*

**Definition** **9.** *A PFG G=(V,A,B) with 4 vertices is a PF path $P_{4}$ graph if its vertices can be label $v_{1},v_{2},v_{3},v_{4}$ such that $\left(\left(v_{i},v_{i}+1\right),\mu_{B},\eta_{B},\nu_{B}\right)\neq \left(0,0,0\right)$, 1≤i≤3.*

**Definition** **10.** *A PFG G=(V,A,B) with 4 vertices $v_{1},v_{2},v_{3},v_{4}$ is a PF matching $2K_{2}$ graph if there are no PF edges adjacent to each other.*

**Definition** **11.** *A configuration with 4 vertices p,q,r,s∈V constitute a PFA4-C if $(\left(p,q\right),\mu_{B},\eta_{B},\nu_{B}$)* *≠(0,0,0) and $(\left(s,r\right),\mu_{B},\eta_{B},\nu_{B})\neq \left(0,0,0\right)$, consequently $(\left(p,r\right),\mu_{B},\eta_{B},\nu_{B})=\left(0,0,0\right)$ and $(\left(q,s\right),\mu_{B},\eta_{B},\nu_{B})=\left(0,0,0\right)$.*

*Based on the degree of TMS, AMS and FMS of the edges (p,s) and (q,r) the PFA4-C may induces 3 types of PF-subgraph:*

(1) *a PF square $C_{4}$ graph if $(\left(p,s\right),\mu_{B},\eta_{B},\nu_{B})\neq \left(0,0,0\right)$ and $(\left(q,r\right),\mu_{B},\eta_{B},\nu_{B})\neq \left(0,0,0\right)$.*
(2) *a PF path $P_{4}$ graph if $(\left(p,s\right),\mu_{B},\eta_{B},\nu_{B})\neq \left(0,0,0\right)$ and $(\left(q,r\right),\mu_{B},\eta_{B},\nu_{B})=\left(0,0,0\right)$;*
*or, $(\left(p,s\right),\mu_{B},\eta_{B},\nu_{B})=\left(0,0,0\right)$ and $(\left(q,r\right),\mu_{B},\eta_{B},\nu_{B})\neq \left(0,0,0\right)$.*
(3) *a PF matching $2K_{2}$ graph if $(\left(p,s\right),\mu_{B},\eta_{B},\nu_{B})=\left(0,0,0\right)$ and $(\left(q,r\right),\mu_{B},\eta_{B},\nu_{B})=\left(0,0,0\right)$.*

**Figure 3:**
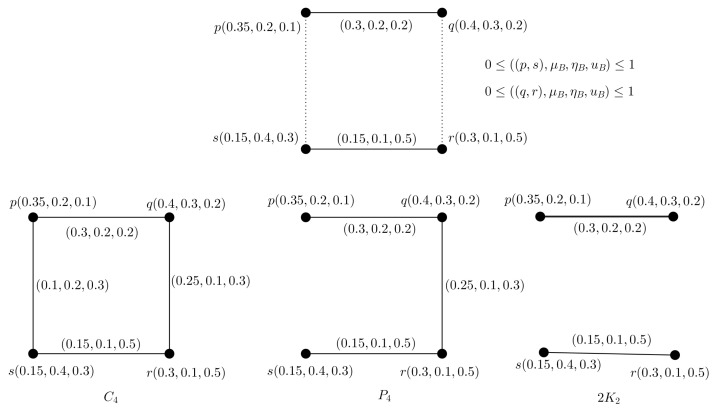
Induced subgraphs of PFA4-C.

**Example** **3.** *Through an example, we depicted PFA4-C, $C_{4}$, $P_{4}$ and $2K_{2}$ shown in Figure 3.*

**Definition** **12.** *A strong PF alternating 4-cycle is a PF alternating 4-cycle if PF square $C_{4}$ graph can be induced from it.*

**Definition** **13.** *A PFS Ⓢ is said to be a stable (independent) set if $(\left(p,q\right),\mu_{B},\eta_{B},\nu_{B})=\left(0,0,0\right)$, ∀p,q∈Ⓢ.*

**Theorem** **1.** *A FTG is a special PFTG.*

**Proof.**  Let $G=(A,B;t_{1})$ be FTG with threshold $t_{1}$ such that $\sum_{p\in Ⓢ}\mu_{A}\left(p\right)\leq t_{1}$ iff Ⓢ is SS in G. For a PFG, we know that $\eta_{A}\left(p\right)=0$, $\nu_{A}\left(p\right)=0$ for all p∈Ⓢ. Take $t_{2}=t_{3}=n$ (*n* is no. of nodes of V), then ∃$t_{i}\geq 0$, i=1,2,3 such that $\sum_{p\in Ⓢ}\mu_{A}\left(p\right)\leq t_{1}$, $\sum_{p\in Ⓢ}\eta_{A}\left(p\right)\leq t_{2}$ and $\sum_{p\in Ⓢ}\nu_{A}\left(p\right)\leq t_{3}$. Thus, a FTG is a PFTG. □

**Theorem** **2.** *A PFTG does not have any PFA4-C.*

**Proof.**  Let $G=(A,B;t_{1},t_{2},t_{3})$ be a PFTG. Suppose it has a PFA4-C. Then ∃p,q,r,s∈V such that $(\left(p,q\right),\mu_{B},\eta_{B},\nu_{B})\neq \left(0,0,0\right)$, $(\left(s,r\right),\mu_{B},\eta_{B},\nu_{B})\neq \left(0,0,0\right)$, $(\left(p,r\right),\mu_{B},\eta_{B},\nu_{B})=\left(0,0,0\right)$ and $(\left(q,s\right),\mu_{B},\eta_{B},\nu_{B})=\left(0,0,0\right)$. Since $G=(A,B;t_{1},t_{2},t_{3})$ is a PFTG, then

(1) $$\mu_{A}\left(p\right)+\mu_{A}\left(q\right)>t_{1},\eta_{A}\left(p\right)+\eta_{A}\left(q\right)>t_{2},\nu_{A}\left(p\right)+\nu_{A}\left(q\right)>t_{3},$$

(2) $$\mu_{A}\left(r\right)+\mu_{A}\left(s\right)>t_{1},\eta_{A}\left(r\right)+\eta_{A}\left(s\right)>t_{2},\nu_{A}\left(r\right)+\nu_{A}\left(s\right)>t_{3},$$

(3) $$\mu_{A}\left(p\right)+\mu_{A}\left(r\right)\leq t_{1},\eta_{A}\left(p\right)+\eta_{A}\left(r\right)\leq t_{2},\nu_{A}\left(p\right)+\nu_{A}\left(r\right)\leq t_{3},$$

(4) $$\mu_{A}\left(q\right)+\mu_{A}\left(s\right)\leq t_{1},\eta_{A}\left(q\right)+\eta_{A}\left(s\right)\leq t_{2},\nu_{A}\left(q\right)+\nu_{A}\left(s\right)\leq t_{3}.$$

Adding (1) and (2), we get

(5) $$\left\{\begin{array}{c} \mu_{A}\left(p\right)+\mu_{A}\left(q\right)+\mu_{A}\left(r\right)+\mu_{A}\left(s\right)>2t_{1},\;\;\\ \eta_{A}\left(p\right)+\eta_{A}\left(q\right)+\eta_{A}\left(r\right)+\eta_{A}\left(s\right)>2t_{2},\;\;\\ \nu_{A}\left(p\right)+\nu_{A}\left(q\right)+\nu_{A}\left(r\right)+\nu_{A}\left(s\right)>2t_{3}.\;\; \end{array}\right\}$$

Adding (3) and (4), we get

(6) $$\left\{\begin{array}{c} \mu_{A}\left(p\right)+\mu_{A}\left(q\right)+\mu_{A}\left(r\right)+\mu_{A}\left(s\right)\leq 2t_{1},\;\;\\ \eta_{A}\left(p\right)+\eta_{A}\left(q\right)+\eta_{A}\left(r\right)+\eta_{A}\left(s\right)\leq 2t_{2},\;\;\\ \nu_{A}\left(p\right)+\nu_{A}\left(q\right)+\nu_{A}\left(r\right)+\nu_{A}\left(s\right)\leq 2t_{3}.\;\; \end{array}\right\}$$

Obviously. Equation (5) contradicts with Equation (6). Hence, a PFTG cannot have any PFA4-C. □

**Definition** **14.** *A PFG is called a picture fuzzy split graph (PFSG) if its node set can be partitioned into a PF clique and a SS.*

**Theorem** **3.** Let G be a PFG with UCG $G^{*}=\left(V,E\right)$. Then $G^{*}$ is SG, if G is PFTG,.

**Proof.**  Let $G=(A,B;t_{1},t_{2},t_{3})$ be a PFTG. We have to show $G^{*}$ is SG, i.e., V can partitioned into a clique and a SS. Assume that in $G^{*}$, L is the greatest clique. Then only leftover to prove V−L is SS.

If V−L is not a SS, then ∃ an arc (p,q)∈V−L such that $(\left(p,q\right),\mu_{B},\eta_{B},\nu_{B})=\left(0,0,0\right)$. Since, L is the largest clique, then ∃ distinct nodes r,s in L such that $(\left(p,r\right),\mu_{B},\eta_{B},\nu_{B})=\left(0,0,0\right)$ and $(\left(q,s\right),\mu_{B},\eta_{B},\nu_{B})=\left(0,0,0\right)$.

This shows that p,q,r,s creates a PFA4-C, it contradicts that G is a PFTG. Hence, V−L is a SS, and $G^{*}$ is a SG. □

**Theorem** **4.** Let G be PFG with UCG $G^{*}$. If G is PFTG, then $G^{*}$ is a TG in crisp sense.

**Proof.**  Let G be PFTG with TV $t=(t_{1},t_{2},t_{3})$, then $\sum_{p\in Ⓢ}\mu_{A}\left(p\right)\leq t_{1}$, $\sum_{p\in Ⓢ}\eta_{A}\left(p\right)\leq t_{2}$ and $\sum_{p\in Ⓢ}\nu_{A}\left(p\right)\leq t_{3}$ iff Ⓢ is a SS in G. Since, $G^{*}$ is UCG of G, then Ⓢ is a SS in $G^{*}$ also. In the sense of crisp graph theory for $G^{*}$, we set non-negative reals $\mu_{A}\left(p\right)$, for the node p∈V, we have $\sum_{p\in Ⓢ}\mu_{A}\left(p\right)\leq t_{1}$. Then $G^{*}$ is a TG. □

Next, we can decomposed a PFTG G into 3 FGs $G_{1}$, $G_{2}$, $G_{3}$ in such a way that FG $G_{i}$ is created by considering *i*th components of the membership value (MV) of vertices and arcs of G, for i=1,2,3.

Now, we present the following theorems.

**Theorem** **5.** *If G be PFTG with TV $t=(t_{1},t_{2},t_{3})$. Then, its decomposed FGs $G_{1}$, $G_{2}$ and $G_{3}$ are also FTGs with TV $t_{1}$, $t_{2}$ and $t_{3}$, respectively.*

**Proof.**  Let G be PFTG with TV $t=(t_{1},t_{2},t_{3})$, then $\sum_{p\in Ⓢ}\mu_{A}\left(p\right)\leq t_{1}$, $\sum_{p\in Ⓢ}\eta_{A}\left(p\right)\leq t_{2}$ and $\sum_{p\in Ⓢ}\nu_{A}\left(p\right)\leq t_{3}$ iff Ⓢ is a SS in G. As, $G_{i}$, $G_{2}$, $G_{3}$ are the decomposed FGs of G and if *r* be a node belongs in $G_{i}$, i=1,2,3 and G. Then, MV of *r* in $G_{1}$, $G_{2}$ and $G_{3}$ are respectively $\mu_{A}\left(r\right)$, $\eta_{A}\left(r\right)$ and $\nu_{A}\left(r\right)$, these are respectively the degree of TMS, AMS and FMS of r∈G.

Now, we have, $\sum_{p\in Ⓢ}\mu_{A}\left(p\right)\leq t_{1}$, $\sum_{p\in Ⓢ}\eta_{A}\left(p\right)\leq t_{2}$ and $\sum_{p\in Ⓢ}\nu_{A}\left(p\right)\leq t_{3}$. This proves that $G_{1}$, $G_{2}$ and $G_{3}$ are the FTGs with TV $t_{1}$, $t_{2}$ and $t_{3}$, respectively. □

**Figure 4:**
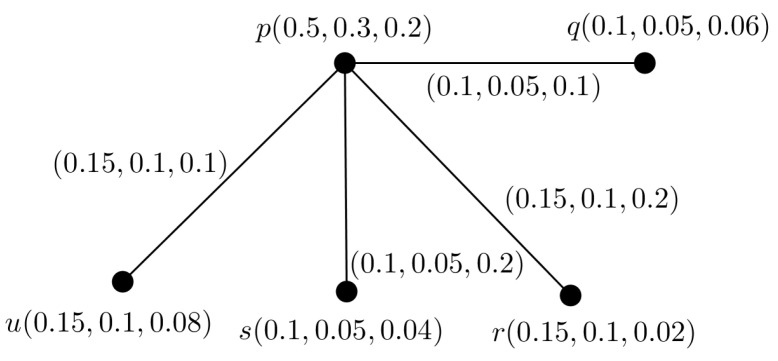
PFTG G.

**Figure 5:**
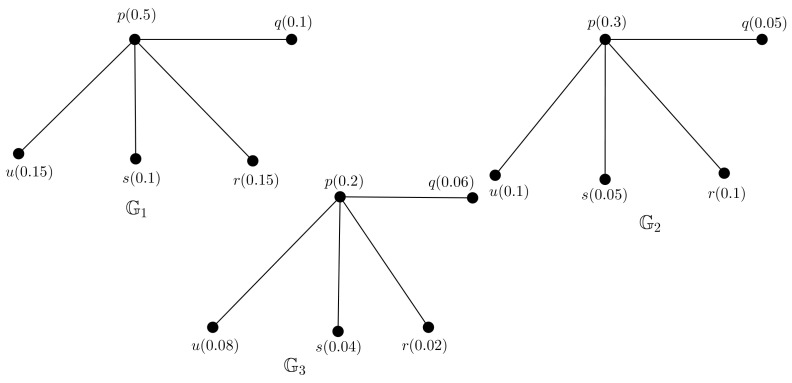
Three FTGs $G_{1},G_{2},G_{3}$.

**Example** **4.** *We consider a PFTG G with TV (0.5,0.3,0.2) shown in Figure 4. We decomposed G into 3 FGs $G_{1}$, $G_{2}$ and $G_{3}$ shown in Figure 5, they are also FTGs with TV 0.5,0.3 and 0.2, respectively.*

**Theorem** **6.** *If $G_{1}$, $G_{2}$, $G_{3}$ are 3 FTGs with TV $t_{1}$, $t_{2}$, $t_{3}$, respectively and whose UCGs are isomorphic. Then their composed PFTG G is also a PFTG with TV $t=(t_{1},t_{2},t_{3})$.*

**Proof.**  Since $G_{i}$, i=1,2,3 is the FTG with TV $t_{i}$, i=1,2,3, we have $\sum_{p\in {Ⓢ}_{1}}\mu_{A}\left(p\right)\leq t_{1}$, $\sum_{p\in {Ⓢ}_{2}}\eta_{A}\left(p\right)\leq t_{2}$ and $\sum_{p\in {Ⓢ}_{3}}\nu_{A}\left(p\right)\leq t_{3}$, where ${Ⓢ}_{i}$ is the SS of $G_{i}$, i=1,2,3. As, $G_{i}^{*}$, the crisp graph of $G_{i}$, are isomorphic for i≠j, (i,j=1,2,3), then the SSs for each FTG remain same, say, Ⓢ and it is the SS of composed PFTG G. Thus, we have $\sum_{p\in Ⓢ}\mu_{A}\left(p\right)\leq t_{1}$, $\sum_{p\in Ⓢ}\eta_{A}\left(p\right)\leq t_{2}$ and $\sum_{p\in Ⓢ}\nu_{A}\left(p\right)\leq t_{3}$. Therefore, G is a PFTG with TV $t=(t_{1},t_{2},t_{3})$. □

**Theorem** **7.** *The TV of a complete PFG is (0,0,0).*

**Proof.**  Suppose G be complete PFG. Then, any two nodes of it are connected. So, the SS in G becomes Ⓢ=∅. then, $\sum_{p\in Ⓢ}\mu_{A}\left(p\right)\leq 0$, $\sum_{p\in Ⓢ}\eta_{A}\left(p\right)\leq 0$ and $\sum_{p\in Ⓢ}\nu_{A}\left(p\right)\leq 0$. Therefore, the TV of a complete PFG is (0,0,0). □

**Theorem** **8.** *Every PFTG is the PFSG.*

**Proof.**  Suppose $G=(A,B;t_{1},t_{2},t_{3})$ be a PFTG. We have to show G is PFSG, i.e., V can partitioned into a clique and a SS. Assume that L is the greatest clique of G. Then only leftover to prove V−L is a SS.

If V−L is not a SS, then ∃ an arc (p,q)∈V−L such that $(\left(p,q\right),\mu_{B},\eta_{B},\nu_{B})=\left(0,0,0\right)$. Since, L is the largest clique, then ∃ distinct nodes r,s∈L such that $(\left(p,r\right),\mu_{B},\eta_{B},\nu_{B})=\left(0,0,0\right)$ and $(\left(q,s\right),\mu_{B},\eta_{B},\nu_{B})=\left(0,0,0\right)$.

This shows that p,q,r,s creates a PFA4-C, it contradicts that G is a PFTG. Hence, V−L is a SS, and G is a PFSG. □

Converse part of the above theorem is given below.

**Theorem** **9.** *Every PFSG is either a PFTG or it can be converted to PFTG after modification of the TMS, AMS and FMS of nodes.*

**Proof.**  Suppose G=(V,A,B) be a PFSG. Then ∃ a PF clique L and a SS V−L. If G is a PFTG, then there is nothing to prove.

If PFSG is not PFTG, then changes can be made to the TMS, AMS and FMS of nodes such that for some TVs $t=(t_{1},t_{2},t_{3})$ the conditions $\sum_{p\in Ⓢ}\mu_{A}\left(p\right)\leq t_{1}$, $\sum_{p\in Ⓢ}\eta_{A}\left(p\right)\leq t_{2}$ and $\sum_{p\in Ⓢ}\nu_{A}\left(p\right)\leq t_{3}$ iff Ⓢ is a SS in G, is hold good. In case, when a node p∈V−L is adjacent to q∈L, then the conditions are not violate. Therefore, a PFSG becomes a PFTG. □

**Theorem** **10.** *A PFTG can be made from a single node PFG by continually adding PF isolated node or a PF dominating node.*

**Proof.**  Let G be a PFG with a single vertex $\left\{p_{0}\right\}$. Theorem 9 states that each PFSG can be formed into a PFTG, it can be proved if after adding a PF isolated node or a PF dominating node the resultant graph is also an PFSG.

Since G is single node PFG, it can be assumed as PFSG with PF clique L=∅ and a SS $Ⓢ=\{p_{0}\}$. Now, a node $p_{1}$ can be added in two ways, either as a PF isolated node or a PF dominating node. If $p_{1}$ is isolated node then $p_{1}\in Ⓢ$ or $p_{1}$ is a dominating node then $p_{1}\in L$. The resultant graph remains a PFSG. Again, we know every PFSG is either a PFTG or it can be converted to PFTG after modification of the TMS, AMS and FMS of nodes. Hence the theorem. □

**Definition** **15.** *The TD t(G) of a PFG G, is the least +ve integer m for which ∃m number of picture fuzzy threshold-subgraphs (PFTSGs) of G, say, $G_{1},G_{2},\ldots ,G_{m}$ covers the edges set (ES) of G. The TD of a PFTG is at least 1.*

**Figure 6:**
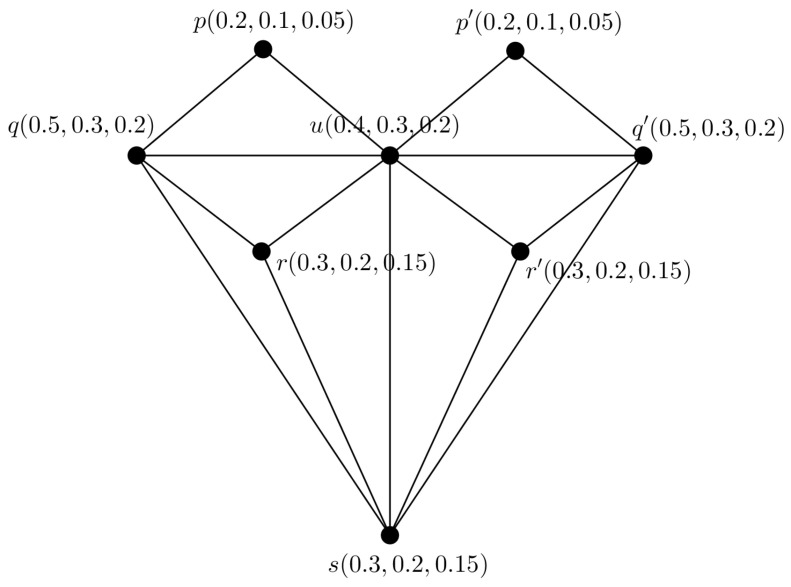
PFG G.

**Figure 7:**
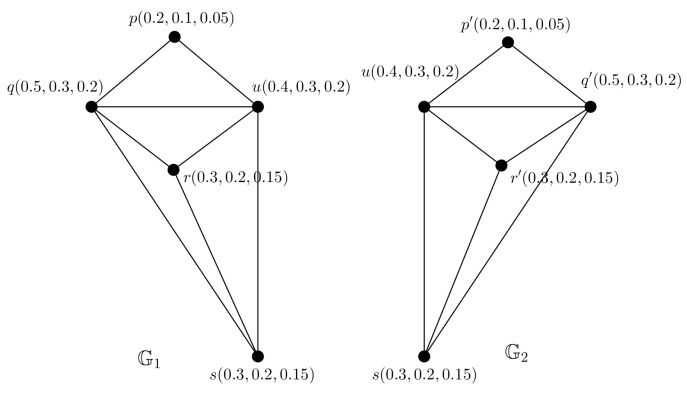
PFTGs $G_{1},G_{2}$.

**Example** **5.** *Consider a PFG G shown in Figure 6. We can construct 2 PFTSGs $G_{1}$ and $G_{2}$ with TV (0.5,0.3,0.2) shown in Figure 7, they covers the ES of G.*

**Theorem** **11.** *The TD of a PFG is at least 1.*

**Proof.**  To show, the TD of a PFG G is non-zero, we have to prove, each PFG has minimum one PFTSG it can cover the ES of G. If G is itself a PFTG then there is nothing to prove. But, if G is not a PFTG. We know, every sole node PFG can made a PFTG. Then, each PFG must have minimum one subgraph which is a PFTG and cover ES of G. Thus, there always exists a TD of any PFG. □

**Definition** **16.** *The α-cut (0≤α≤1) of a PFG G=(V,A,B) is $G_{\alpha }=\left(A_{\alpha },B_{\alpha }\right)$ such that $A_{\alpha }=\left\{p\in V:\mu_{A}\left(p\right)\geq \alpha ,\eta_{A}\left(p\right)\geq \alpha ,\nu_{A}\left(p\right)\geq \alpha \right\}$ and $B_{\alpha }=\left\{\left(p,q\right):\mu_{B}\left(p,q\right)\geq \alpha ,\eta_{B}\left(p,q\right)\geq \alpha ,\nu_{B}\left(p,q\right)\geq \alpha \right\}$.*

**Theorem** **12.** *The α-cut (0≤α≤1) of a PFTG is also a PFTG.*

**Proof.**  Let G be PFTG with TV $t=(t_{1},t_{2},t_{3})$, then $\sum_{p\in Ⓢ}\mu_{A}\left(p\right)\leq t_{1}$, $\sum_{p\in Ⓢ}\eta_{A}\left(p\right)\leq t_{2}$ and $\sum_{p\in Ⓢ}\nu_{A}\left(p\right)\leq t_{3}$ iff Ⓢ is SS in G. Let $G_{\alpha }=\left(A_{\alpha },B_{\alpha }\right)$ be the α-cut of G such that $A_{\alpha }=\left\{p\in V:\mu_{A}\left(p\right)\geq \alpha ,\eta_{A}\left(p\right)\geq \alpha ,\nu_{A}\left(p\right)\geq \alpha \right\}$ and $B_{\alpha }=\left\{\left(p,q\right):\mu_{B}\left(p,q\right)\geq \alpha ,\eta_{B}\left(p,q\right)\geq \alpha ,\nu_{B}\left(p,q\right)\geq \alpha \right\}$.

Case:I When in $G_{\alpha }$ the no. of nodes remains same with G but the no. of arcs may decreases. Let ${Ⓢ}_{1}$ be the SS in $G_{\alpha }$. The cardinality of ${Ⓢ}_{1}$ never decreases than Ⓢ, i.e., ${Ⓢ}_{1}$ may contains more nodes of G along with the nodes of Ⓢ. Then we have $\sum_{p\in {Ⓢ}_{1}}\mu_{A}\left(p\right)\leq t_{1}^{*}$, $\sum_{p\in {Ⓢ}_{1}}\eta_{A}\left(p\right)\leq t_{2}^{*}$ and $\sum_{p\in {Ⓢ}_{1}}\nu_{A}\left(p\right)\leq t_{3}^{*}$ iff ${Ⓢ}_{1}$ is a SS in $G_{\alpha }$, where we choose suitable TV $t^{*}=\left(t_{1}^{*},t_{2}^{*},t_{3}^{*}\right)$ for which the threshold conditions are satisfied.

Case:II When in $G_{\alpha }$ the no. of nodes and arcs may decreases than G. The cardinality of ${Ⓢ}_{1}$ decreases than Ⓢ, i.e., ${Ⓢ}_{1}$ may contains more nodes of G along with the nodes of Ⓢ. Then $\sum_{p\in {Ⓢ}_{1}}\mu_{A}\left(p\right)\leq t_{1}$, $\sum_{p\in {Ⓢ}_{1}}\eta_{A}\left(p\right)\leq t_{2}$ and $\sum_{p\in {Ⓢ}_{1}}\nu_{A}\left(p\right)\leq t_{3}$ iff ${Ⓢ}_{1}$ is a SS in $G_{\alpha }$.

Therefore, $G_{\alpha }$ becomes a PFTG in all cases. □

**Theorem** **13.** *The α-cut of a PFG has TD at least 1.*

**Proof.**  Let G be a PFG with UCG $G^{*}=\left(V,E\right)$. Assume that $G_{\alpha }$ be the α-cut of G. We have to show ∃ minimum one PFTSG which covers the ES of $G_{\alpha }$.

If $G_{\alpha }$ is a PFTG then $G_{\alpha }$ itself fulfill the conditions. Then, $G_{\alpha }$ has TD 1.

If $G_{\alpha }$ is not a PFTG, we have by Theorem 10, every single vertex PFG can made a PFTG. Hence, each PFG must have minimum one subgraph which is a PFTG and cover ES of $G_{\alpha }$. Therefore, the α-cut of a PFG has TD at least 1. □

**Theorem** **14.** *For any PFG, G the TD t(G)≤|V|−α(G). If G is without triangle, then t(G)=|V|−α(G), where α(G) is the cardinality of the greatest SS of G.*

But, if t(G)=|V|−α(G), then G need not be triangle free. It can discussed by an example.

**Figure 8:**
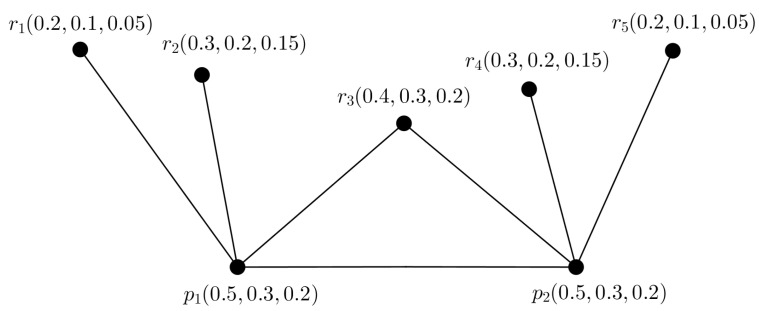
PFG G.

**Figure 9:**
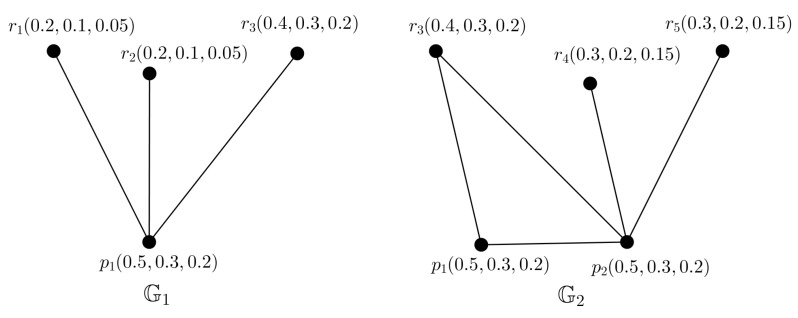
Two PFTGs $G_{1},G_{2}$.

**Example** **6.** *Consider a PFTG G shown in Figure 8. Here, the largest SS is $\left\{r_{1},r_{2},\ldots ,r_{5}\right\}$. Then picture fuzzy TD of G is t(G)=2, as minimum two PFTSGs (shown in Figure 9) are needed to cover the ES of G. Thus, ∣V∣−α(G)=t(G) is satisfied. But, we see that the nodes $p_{1},p_{2},r_{3}$ makes a triangle in G.*

**Theorem** **15.** *If PFG G has no triangle, then $t_{p}\left(G\right)=t\left(G\right)$.*

**Proof.**  Let G be PFG without triangle. The TD t(G) of G is the least no. of PFTSGs which are needed to cover ES of G. If more than one PFTSGs contains a strong arc, then we eject it from all PFTSGs except one PFTSG. Then such PFTSGs cover ES of G without any common arc. Hence, $t_{p}\left(G\right)=t\left(G\right)$. □

But, if $t_{p}\left(G\right)=t\left(G\right)$, then G need not be triangle free. It can discussed by an example.

**Figure 10:**
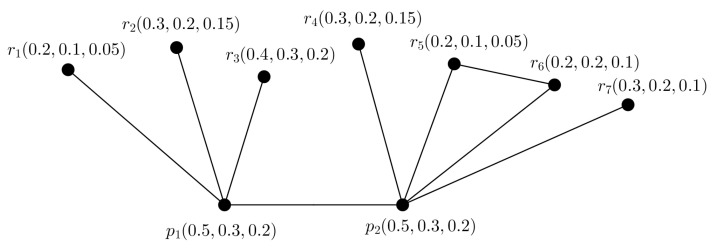
PFG G.

**Figure 11:**
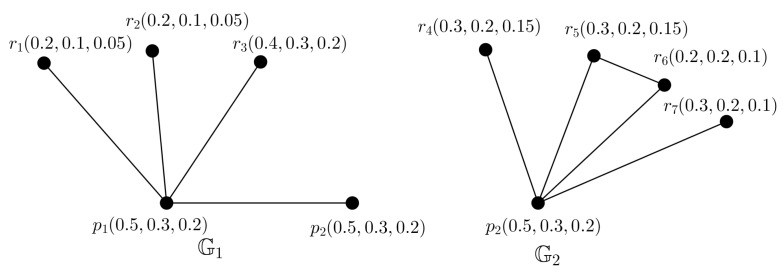
Two PFTSGs $G_{1},G_{2}$.

**Example** **7.** *Consider a PFTG G with nine nodes shown in Figure 10. Here, we can obtain two PFTSGs without any common arcs (shown in Figure 11), those cover the ES of G. Hence, $t\left(G\right)=t_{p}\left(G\right)=2$. But, we see that the nodes $p_{2},r_{5},r_{6}$ makes a triangle in G.*

**Definition** **17.** *The PN $t_{p}\left(G\right)$ of a PFG G, is the least +ve integer p for which *∃*p no. of PFTSGs of G, say, $G_{1},G_{2},\ldots ,G_{p}$ covers the ES of G and they have no common edges.*

**Theorem** **16.** *The TD of a PFG and its decomposed FGs are same.*

**Proof.**  Let G be PFG with TV $t=(t_{1},t_{2},t_{3})$ and $G_{1},G_{2},G_{3}$ be its decomposed FGs. Let t(G)=m. Then, there is *m* PFTSGs which cover ES of G without any common arcs.

Now, the decomposed FGs $G_{k}$, k=1,2,3 have an ES, same with the ES of G. Thus, the ES of $G_{k}$ can also be covered by an equal no. of FGs. Hence, $t\left(G_{k}\right)=m=t\left(G\right)$, k=1,2,3. □

**Theorem** **17.** *The PN of a PFG and its decomposed FGs are same.*

**Proof.**  Let G be PFG with TV $t=(t_{1},t_{2},t_{3})$ and $G_{1},G_{2},G_{3}$ be its decomposed FGs. Let $t_{p}\left(G\right)=p$. Then, there is *p* PFTSGs which cover ES of G without any common arcs.

Now, the decomposed FGs $G_{k}$, k=1,2,3 have an ES, same as the ES of G. Thus, the ES of $G_{k}$ can also be covered by an equal no. of FGs without any common arc. Hence, $t_{p}\left(G_{k}\right)=p=t_{p}\left(G\right)$, k=1,2,3. □

## 5. An Application of Picture Fuzzy Threshold Graphs in Medicine Replenishment Problem

The PFTG is an important mathematical model to represent the information in many real connected graphical systems, in which the vertices and edges both lie under PF information. In the following, we mainly discuss about the application of PFTG in resource allocation problem to control medicine resource.

### 5.1. Model Construction

Now a days, human life closely depends on medicine. It is the world’s most crucial essential in present day scenario. Several types of medicines are used in our daily life like as generic medicines, homeopathic medicines, herbal medicines, etc. It is really significant for the pharmaceutical firms to fulfill human demand and supply sufficient amount of medicines in the market within minimal cost.

**Figure 12:**
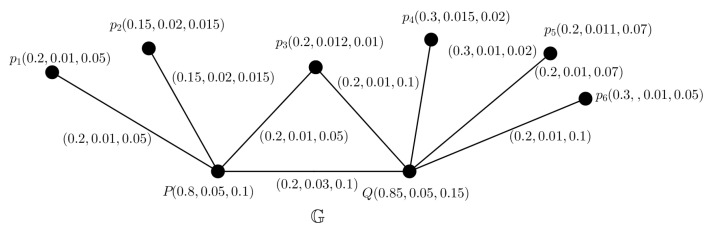
PFG G.

Let us consider 2 resources of medicine say, pharmaceutical firms *P* and *Q*, and suppose there are 6 states $p_{i},i=1,2,\ldots ,6$, which are connected to these firms for getting sufficient medicines. Pharmaceutical sectors supply medicines through transport in all the states. There may arise some problems like communication failure, insufficient infrastructure, damage of medicines, pressure of global demand, etc. All of these issues, affect the amount of medicine needed in each state. The framework of medicine resources and states are modelled by a PFG G as shown in Figure 12, where pharmaceutical firms and states are represented by vertices and there exists an edge between two vertices if one can supply medicine to another connecting the firms and states. Each of them has degree of TMS, AMS and FMS. The total amount of medicine to be supplied varies day to day from 2 firms. Here, we will find out the minimum amount of medicine to be supplied from each pharmaceutical firms so that the medicine supply could fulfil the actual demand of states by using picture FTG model. We can not ignore the damage of medicines during transport from the pharmaceutical firms to the states. MVs of the pharmaceutical firms are denoted as the degree of supply amount, indeterminacy of supply amount and minimal storage amount of medicines of the firms. MVs of cities are denoted as the degree of actual consumption amount, indeterminacy of consumption amount and damage amount of medicines of the state. The edge MVs between firms and states are denoted as no damage amount, indeterminacy of damage amount and damage amount of medicines during transport from firms to states. The MVs between two pharmaceutical firms are denoted as the degree of supply amount, indeterminacy of supply amount and no supply amount of medicines between two firms.

### 5.2. Decision Making

**Figure 13:**
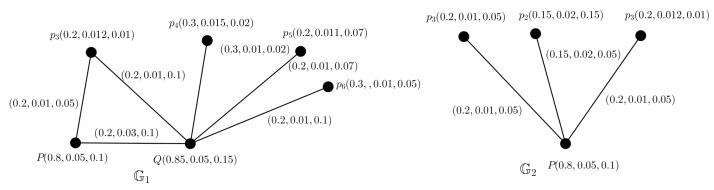
Two PFTSGs $G_{1},G_{2}$.

Since the amount of medicine consumption of each state is dominated by 2 pharmaceutical firms, therefore TD is 2. Since, the maximum SS in G is $\{p_{i},i=1,2,\ldots ,6$}, then PN is at most 2 by using Theorem 14. Then, we can induce 2 PFTSGs $G_{1}$ and $G_{2}$ (shown in Figure 13) from this PFG.

In $G_{1}$, the maximum SS is ${Ⓢ}_{1}=\left\{p_{3},p_{4},p_{5},p_{6}\right\}$ for the SS ${Ⓢ}_{1}$, we have

$\sum_{p\in {Ⓢ}_{1}}\mu_{A}\left(p\right)=0.2+0.3+0.2+0.3=1\leq 1$

$\sum_{p\in {Ⓢ}_{1}}\eta_{A}\left(p\right)=0.012+0.015+0.011+0.01=0.048\leq 0.048$

$\sum_{p\in {Ⓢ}_{1}}\nu_{A}\left(p\right)=0.01+0.02+0.07+0.05=0.15\leq 0.15$.

Therefore, the TV for $G_{1}$ is (1,0.048,0.15).

In $G_{2}$, the maximum SS is ${Ⓢ}_{2}=\left\{p_{1},p_{2},p_{3}\right\}$ and for the SS ${Ⓢ}_{2}$, we have

$\sum_{p\in {Ⓢ}_{2}}\mu_{A}\left(p\right)=0.2+0.15+0.2=0.55\leq 0.55$

$\sum_{p\in {Ⓢ}_{2}}\eta_{A}\left(p\right)=0.01+0.02+0.012=0.042\leq 0.042$

$\sum_{p\in {Ⓢ}_{2}}\nu_{A}\left(p\right)=0.05+0.015+0.01=0.075\leq 0.075$.

Therefore, the TV for $G_{2}$ is (0.55,0.042,0.075).

Degree of TMS of each node denotes the actual consumption of medicine of the state. From the TV of $G_{1}$, we see that the pharmaceutical firms *P* and *Q* need minimum 1 amount of medicine for actual consumption for $p_{i},i=3,4,\ldots ,6$. Here each single pharmaceutical firm can not supply whole medicines among $p_{i},i=3,4,\ldots ,6$ as they need minimum 1 amount of medicine for actual consumption and any vertices, namely *P* and *Q* can not have MV greater than 1. From the TV of $G_{2}$, we see that the pharmaceutical firm *Q* demands minimum 0.55 quantity of medicine for actual consumption of $p_{i},i=1,2,3$. Hence, we can conclude that together the pharmaceutical firms *P* and *Q* can supply sufficient medicines for the states.

Based on the above discussion, we observed that picture FTG really plays a vital role to control medicine resources. In addition, we also acknowledge that picture FTG models are more appropriate and beneficial than the other TG models in controlling medicine resource, because PFSs are more effective to deal with uncertainty and vagueness compared to fuzzy sets and IF sets.

## 6. Comparative Study with Existing Methods

In existing papers, the information was taken as interval-valued fuzzy, IF and *m*-polar fuzzy sense to solve resource allocation problems. When more possible types of vagueness and uncertainty grow in information then the existing methods are not suitable to handle such information. In these scenarios, the information should be taken as PF sense to model the design instead of interval-valued fuzzy, IF and *m*-polar fuzzy sense.

Pramanik et al. [13] proposed a TG model to control the flow of information with interval-valued fuzzy information. In interval-valued fuzzy sets, only membership values are considered. Yang and Mao [2] introduced an intuitionistic FTG model and used to control water resource and power resource. In intuitionistic FTG models, only membership and non-membership values of vertices and edges are considered. Mahapatra and Pal [16] studieded *m*-polar FTG model in which each vertex and edge has a *m* number of membership values and implemented it on a resource power controlling system.

So these models are not applicable when the models are considered in other environment like in PF environment. In our currently developed PFTG models, we include another parameter called neutral membership value and it is practically useful in case of real life problem. The PFTG models are more generalized and superior than the above FTG models. Moreover, it will be capable to accommodate more vagueness and uncertainties and provide better results than the existing models. So, our study is the extension of the study of the above methods.

## 7. Conclusions

In the resource environment, many researchers have proposed various TG models for improving the performance of the resource controlling system. This paper begins by studying and understanding several aspects of TG models in PF environment. In the literature survey, various methods were identified and studied. Even though many researchers proposed various models, it is found that there is no efficient and effective model that gives a combined solution for many issues. This research proposed an efficient and an effective TG models in which various issues, such as to deal with the system having more vagueness and uncertainties, and an indeterminate information, are addressed by considering an extra parameter ’neutrality’.

In general, the main contributions of this work can be highlighted as follows: (1) Proposing a new adaptive PFTG model along with its several properties to improve fault of existing models in different environment. (2) We introduce picture fuzzy TD and picture fuzzy PN and investigated relations between them and, studied some of their properties on decomposed PFTG. (3) We have proved that under some conditions, a PFG is equivalent to an PFTG. Some other properties related to PFTGs have also been studied in this paper. (4) Especially, we have obtained that the underlying graph of each PFG is a split graph, and conversely, for a given split graph can be converted to an PFTG. (5) Finally, a numerical example with its comparison against existing rival approaches are provided to demonstrate the characteristics of the proposed models. (6) It has been shown that PFTG model can be used to control medicine resource, and also PFTGs are more appropriate than the existing TG models in controlling medicine resource.

The main limitations, inherent in this approach, is that (i) This work mainly focuses on PFTG. (ii) It should be noted that our model is limited by the fact that we have neglected many other effects that could lead to system collapse (traffic congestion, loss of vehicles, etc.). However, our analysis, by not taking into account all of these additional factors, provides a conservative limit when investigating a possible real collapse. (iii) If membership values of the characters are given in different interval-valued PF environments, then PFTG model cannot be used. (iv) Here, only one resource is included; hence, the descriptive outcome of the study cannot be generalized. (v) This types of proposed models are mainly used in controlling system.

Even though this study proposes a novel approach to control resource allocation problems, there are still some new research topics which are needed to be further studied by applying this concept. Some of them are mentioned below: (1) Applying the proposed model to a real-time environment. (2) Our research work will be extended depending on PFTG to find some more characteristics along with some applications. (3) This work will be done on application of PFTGs in set packing problems, and we will propose an efficient algorithms to search the PFTGs from PFGs. (4) Besides, we will further discuss about the relationship between PFTGs and its IF independent sets, and try to do a deep research on the PFTGs theory. (5) The proposed model is tested with only one resource. In the future, the number of resources may be increased and tested as an extension of the proposed models.

## Data Availability

Not applicable.

## Abbreviations

GT	Graph theory
IF	Intuitionistic fuzzy
PF	Picture fuzzy
PFS	Picture fuzzy set
PFG	Picture fuzzy graph
PFA4-C	Picture fuzzy alternating 4-cycle
FG	Fuzzy graph
SS	Stable set
ES	Edges set
TG	Threshold graph
SG	Split graph
TV	Threshold value
TD	Threshold dimension
PN	Partition number
MV	Membership value
UCG	Underlying crisp graph
FTG	Fuzzy threshold graph
PFTG	Picture fuzzy threshold graph
PFSG	Picture fuzzy split graph
PFTSG	Picture fuzzy threshold-subgraph
TMS, AMS and FMS	Truth, abstinence and false membership value, respectively.
